# Acquisition, Replication and Inoculation of *Candidatus* Liberibacter asiaticus following Various Acquisition Periods on Huanglongbing-Infected Citrus by Nymphs and Adults of the Asian Citrus Psyllid

**DOI:** 10.1371/journal.pone.0159594

**Published:** 2016-07-21

**Authors:** El-Desouky Ammar, John E. Ramos, David G. Hall, William O. Dawson, Robert G. Shatters

**Affiliations:** 1 United States Department of Agriculture-Agricultural Research Service, Horticultural Research Laboratory, Fort Pierce, Florida, United States of America; 2 University of Florida, IFAS-CREC, Lake Alfred, Florida, United States of America; Volcani Center, ISRAEL

## Abstract

The Asian citrus psyllid, *Diaphorina citri* (Hemiptera: Liviidae), is the primary vector of *Candidatus* Liberibacter asiaticus (Las) implicated as causative agent of citrus huanglongbing (citrus greening), currently the most serious citrus disease worldwide. Las is transmitted by *D*. *citri* in a persistent-circulative manner, but the question of replication of this bacterium in its psyllid vector has not been resolved. Thus, we studied the effects of the acquisition access period (AAP) by nymphs and adults of *D*. *citri* on Las acquisition, multiplication and inoculation/transmission. *D*. *citri* nymphs or adults (previously non-exposed to Las) were caged on Las-infected citrus plants for an AAP of 1, 7 or 14 days. These ‘Las-exposed’ psyllids were then transferred weekly to healthy citrus or orange jasmine plants, and sampled via quantitative polymerase chain reaction (qPCR) analysis 1–42 days post-first access to diseased plants (padp); all tested nymphs became adults 7–14 days padp. Our results indicate that following 1 or 7 day AAP as nymphs 49–59% of Las-exposed psyllids became Las-infected (qPCR-positive), whereas only 8–29% of the psyllids were infected following 1–14 day AAP as adults. Q-PCR analysis also indicated that Las titer in the Las-exposed psyllids (relative to that of the psyllid S20 ribosomal protein gene) was: 1) significantly higher, and increasing at a faster rate, following Las acquisition as nymphs compared to that following Las acquisition as adults; 2) higher as post-acquisition time of psyllids on healthy plants increased reaching a peak at 14–28 days padp for nymphs and 21–35 days padp for adults, with Las titer decreasing or fluctuating after that; 3) higher with longer AAP on infected plants, especially with acquisition as adults. Our results strongly suggest that Las multiplies in both nymphs and adults of *D*. *citri* but attains much higher levels in a shorter period of time post-acquisition when acquired by nymphs than when acquired by adults, and that adults may require longer access to infected plants compared to nymphs for Las to reach higher levels in the vector. However, under the conditions of our experiments, only *D*. *citri* that had access to infected plants as nymphs were able to inoculate Las into healthy citrus seedlings or excised leaves. The higher probability of Las inoculation into citrus by psyllids when they have acquired this bacterium from infected plants during the nymphal rather than the adult stage, as reported by us and others, has significant implications in the epidemiology and control of this economically important citrus disease.

## Introduction

Huanglongbing (HLB), or citrus greening, is currently the most destructive citrus disease in Florida USA and many other citrus growing areas around the world [1,2,3]. The HLB disease bacterium can infect all commercial citrus cultivars and may cause substantial economic losses by promoting fruit drop, rendering fruit inedible or unmarketable, and shortening the lifespan of infected trees [4,5,6]. Yield reduction may reach 30–100% depending on the proportion of the canopy affected and the age of trees during inoculation [2]. Three closely related, phloem limited ά-proteobacteria are associated with this disease in various parts of the world: *Candidatus* Liberibacter asiaticus (from Asia and the Americas), *Candidatus* Liberibacter africanus (from Africa) and *Candidatus* Liberibacter americanus (from Brazil) [6,7]. Although Koch’s postulates remain to be completed, mainly because these bacteria are so far non-culturable, Tyler et al. [8] provided evidence using phloem metagenomic DNA which strongly suggested that *Candidatus* Liberibacter asiaticus (Las) is the main, if not the only, phloem microbe present in plants with severe HLB symptoms in the USA.

The Asian citrus psyllid *Diaphorina citri* Kuwayama (Hemiptera: Liviidae) is the principal insect vector of *Candidatus* Liberibacter spp. in Asia, Brazil and USA, whereas the African psyllid, *Trioza erytreae* (Del Guercio) (Triozidae), is the main vector in African countries, Mauritius, and Re´union [1,2,9]. Some of the transmission characteristics and parameters of the Las bacterium by *D*. *citri* have been studied earlier but several gaps still exist [10–17]. *D*. *citri* can acquire the pathogen within 5–7 h of feeding on diseased plants [1], but only 40% of adults tested positive for the pathogen, using PCR tests, after feeding for 35 days on diseased citrus [16]. *D*. *citri* individuals that acquired Las only during the adult stage were poor vectors of the pathogen, unlike adults that acquired the pathogen as nymphs [16,18]. A latent period of 1–25 days may be required before *D*. *citri* adults can transmit the pathogen after acquisition from diseased citrus, but adults developing from nymphs that acquired the pathogen can inoculate Las into host plants as soon as they emerge from the last nymphal instar [15,16,19,20]. *D*. *citri* adults can remain inoculative (infective) with Las throughout their life after acquiring the pathogen as nymphs [15,19]. Normally, however, low percentages of Las-exposed *D*. *citri* individuals have been reported to be able to inoculate Las into healthy citrus seedlings (1.3–12.2%), although much higher percentages of these psyllids (up to 90%) may prove Las-positive in PCR tests [11,14,16,17]. Thus, it has been suggested that transmission barriers for Las in *D*. *citri* organs may be involved, especially in the midgut and salivary glands [13,14].

Using qPCR and/or fluorescent *in situ* hybridization (FISH), we earlier reported that Las was found in most of the internal organs and tissues of Las-exposed *D*. *citri*, and that Las titer in the midgut and salivary glands was higher than that in other tissues suggesting that Las either accumulates or multiplies in these organs [13,14]. The almost systemic infection of Las in organs and tissues of *D*. *citri* is similar to that of propagative plant pathogens that are known to multiply in their hemipteran vectors [21,22,23]. The question of replication of Las in its vector, however, remains largely unresolved, since the only report on it, by Inoue et al. [18], suggested that Las multiplies in *D*. *citri* when acquired by nymphs but not when acquired by adults. They also suggested that multiplication of the bacterium within the vector is essential for effective transmission since *D*. *citri* that acquired Las as adults did not seem to transmit it whereas those that acquired it as nymphs did. Verification of the role of Las replication in transmission by the psyllid and developing a more in-depth understanding of transmission events and parameters are important in providing characteristics of this pathosystem, thereby facilitating the development of new management strategies targeting pathogen transmission by the vector.

In Inoue’s study of Las replication in *D*. *citri* [18], only 1-day acquisition access period (AAP) on infected citrus plants by nymphs or adults was tested. In the present work, we used qPCR to compare the relative titer of Las in *D*. *citri* individuals that had various AAP’s (from 1 to 14 days) as nymphs or adults on Las-infected citrus. We report that Las appears to multiply in both nymphs and adults, but the latter may require longer access to infected plants compared to nymphs for Las to reach higher levels in the vector.

## Materials and Methods

### Psyllids and Plants Used

We used nymphs or adults of *D*. *citri* from a healthy (non Las-exposed) laboratory colony established during the year 2000 that has been maintained on healthy orange jasmine [*Murraya paniculata* (L.) Jack] trees, and more recently on healthy citrus trees (*Citrus macrophylla* Wester) in the greenhouse as described earlier [24]. No wild *D*. *citri* were introduced into this colony, and individuals from the colony were PCR-assayed every three months to ensure that the colony remained Las-free. Young Las-infected ‘Schaub’ rough lemon trees (*Citrus jambhiri* Lush.), that had been proven to be Las-positive by qPCR, were used for Las acquisition by *D*. *citri* nymphs or adults for the acquisition access periods (AAPs) indicated below. During these AAPs, healthy seedlings of ‘Schaub’ rough lemon were used for feeding healthy control psyllids. Following each AAP treatment, healthy seedlings of sweet orange [*Citrus sinensis* (L.) Osbeck var. *‘*Ridge Pineapple’], or healthy seedlings of orange jasmine were used as ‘holding plants’ to maintain the psyllids of all treatments as mentioned below. Additionally, excised medium-size leaves (~3–4 cm in width and ~6–8 cm in length) or seedlings of sweet orange were used to test the inoculativity of psyllids (their ability to inoculate Las) as described below.

### Las Acquisition and Inoculation by *D*. *citri* Nymphs and Adults

Healthy (non Las-exposed) *D*. *citri* nymphs (3^rd-^4^th^ instars) or adults (<7 d post last molt) were caged on Las-infected lemon flush for various AAP treatments in large Plexiglas tube cages (15 cm diam., 60 cm high, with a nylon screen mesh covered top and 2 mesh ventilation holes). In experiments using nymphs (expts. 1 and 3) only 1- and 7-day AAP treatments were tested (400–600 nymphs/treatment/experiment), whereas with adults (expts. 2 and 4) three AAP treatments of 1, 7, and 14-days were tested (200–300 adults/treatment/expt.). In each experiment, healthy (non Las-exposed) nymphs or adults of a similar age were caged on healthy lemon flush for 7 or14 days, respectively, as control.

At the end of each AAP, Las-exposed and unexposed control psyllids (nymphs or adults) were transferred weekly in smaller groups (≤ 20 insects/plant) to healthy ‘holding plants’ in Plexiglas tube cages (3.8 cm diam., 30 cm high, with a nylon screen mesh covered top and 4 ventilation holes) until most of them died 35 to 42 days post-first access to diseased plants (padp). In the first set of experiments with nymphs and adults (expts. 1 and 2, respectively), the holding plants were young healthy sweet orange seedlings. However, in the second set of experiments (expts. 3 and 4 for nymphs and adults, respectively) healthy orange jasmine seedlings were used for holding the psyllids post acquisition. Previous work had shown that orange jasmine plants are more resistant than sweet orange to Las infection [25], which made it less likely that psyllids would re-acquire Las from these holding plants following the AAP’s tested. Also at the end of each AAP, 3 leaves (from top, middle and lower parts of the plant) from each infected lemon plant used as a source for Las-acquisition were tested by qPCR (all proved Las-positive, with Ct values ranging between 24.52 and 37.71). Healthy control lemon plants used for the control psyllids were also tested by qPCR (all proved Las-negative).

Following each AAP treatment, *D*. *citri* nymphs or adults were sampled for qPCR (12–20 insects/treatment/date/experiment) at the following times: a. at the end of the AAP, with 3–4 h of starvation in a glass vial at room temperature to help clear their gut lumen from ingested Las; b. 1 day post-AAP, during which the psyllids were fed on healthy excised citrus leaves for more thorough clearing of their gut lumen from Las; and c. at weekly intervals from day 0 (first day of AAP) after that. Each group of psyllids/sample (with id. no. and date) were collected in one glass vial and frozen at– 80°C, then processed singly for qPCR as described below. However, for Las titer calculations, only the psyllids that fed on healthy leaves or plants for at least 1 day following the AAP were considered to minimize the risk of including Las bacteria ingested from Las-infected plants immediately post-AAP.

To test their inoculativity with Las, following each AAP treatment, a group of psyllids was caged weekly on fresh healthy sweet orange seedlings or excised leaves (5–6 insects/ seedling or excised leaf/ week; 5–10 seedlings or excised leaves/treatment/week). In these consecutive weekly tests, psyllids that died every week from each group were replaced in the following week by other psyllids from the same treatment to keep the number of insects nearly constant per plant or excised leaf. These inoculativity tests were continued weekly until 35 days padp. It should be noted, however, that all the nymphs used for Las acquisition became adults at 7–14 days padp.

Throughout each experiment, psyllids were kept in a growth chamber at 25.7 ± 0.4°C with 14 h of light per day (RH = 75%). After transferring the psyllids from the sweet orange seedlings weekly, these seedlings (each labeled with its group id. no. and dates) were moved to the greenhouse and sprayed with pesticides (to kill any remaining psyllids). In the greenhouse, these seedlings were observed for symptoms and sampled for qPCR, 3, 6 and 9–12 months later. Inoculated excised leaves, however, were incubated for 7 days at 25°C before being processed for qPCR [26]. All seedlings or excised leaves fed on by the psyllids were tested for Las by qPCR as described below.

### DNA Extraction and qPCR for Psyllids

Crude DNA samples (whole psyllid DNA) were prepared using a crude DNA isolation procedure previously described [14]. The only exception to the published procedure was the addition of four 1.4 to 1.6 mm zirconium silicate sintered beads (Ceroglass, Inc. Columbia, TN) to the tube containing a single psyllid and 150μL lysis buffer (5.32mM KCl, 5.32mM Tris pH 8.4, 47% (v/v) Tween 20, 47% (v/v) NP40) prior to homogenization by Genogrinder (1600RPM for 4 min.). A thermocycler was used to heat samples for 5 min. at 95°C, then cool them for 10 min. at 4°C, followed by centrifugation at 14kRCF for 2 min. 80μL of supernatant was used as template in qPCR. The following primer set amplifying a region of the Las 16s rDNA (Genbank accession #DQ673424) was used to determine Las titer: USHRL-CL1f:5’- CTTACCAGCCCTTGACATGTATAGGA -3’, and USHRL-CL1r:5’- TCCCTATAAAGTACCCAACATCTAGGTAAA -3’. The following primer set amplifying a region of the *D*. *citri* Ribosomal S20 (RPS20) psyllid gene (Genbank accession #DQ673424) was used as an internal reference to quantify psyllid cell number in each reaction: Dci-S20-L:5’- GCCCAAGGGCCCAATCA -3’, and Dci-S20-R:5’- GGAGTCTTACGGGTGGTTATTCTG-3’ [14,27]. Each 25μL reaction consisted of the following reagents: 12.5μL of GoTaq® Master Mix (Promega, Madison WI), 2μL of Primer mix (F/R), and 5μL Crude Psyllid DNA. Cycling parameters consisted of: hold at 95°C for 2 min., 50 cycles (of 95°C for 15 sec., 60°C for 30 sec., 72°C for 30 sec.), and an HRM melt at 74–88°C, hold 1 sec. on 1^st^ step and 2 sec. on each subsequent step next. Two samples from each insect were analyzed with qPCR in two independent runs, and the average Ct value of the two readings was used to determine if each was Las-positive. The relative titer of Las genomes (relative to the RPS20 psyllid gene) was determined using a Delta Ct method [28].

### qPCR on Citrus Leaves and Citrus Seedlings Fed on by *D*. *citri*

To prepare them for qPCR, excised citrus leaves on which *D*. *citri* were fed for an inoculation access period (IAP) of 1 week, as well as leaves sampled from citrus plants (3 leaves/plant) that were similarly fed on by psyllids for 1-week IAP, were washed thoroughly in RNase-Away (Molecular BioProducts, Inc., San Diego, CA) and subsequently rinsed with DI water. This was done to remove *D*. *citri* honeydew excretions, eggs or nymphs, and any Las bacteria that may have been present on the leaf surface [26]. Each leaf midrib was separated from the leaf blade and immediately chopped into very small pieces with a new sterile razor blade. Each sample was then placed in an individual tube that was subsequently stored in a freezer at -80°C until further processing. DNA extraction and qPCR analysis on these leaves were performed as described earlier [26] using HLBaspr primer set targeting the 16S DNA of LAS (5′→3′ sequences: forward TCGAGCGCGTATGCAATACG and reverse GCGTTATCCCGTAGAAAAAGGTAG, probe AGACGGGTGAGTAACGCG with 6-carboxyflourescein reporter dye on the 5′ end and TAMRA quencher on the 3′ end) [29].

### Statistical Analysis

#### Proportion of Las-exposed *D*. *citri* testing Las positive in qPCR and proportion of Las-infected plants/leaves

Effects of the following independent variables, and their interactions, on the proportion of *D*. *citri* testing qPCR-positive for Las were studied using a factorial analyses of variance (ANOVA, PROC GLM, SAS Institute 2010) [30]: AAP (acquisition access period), life stage during AAP (nymph or adult), and time post-acquisition (days padp). The results of all four experiments (two/life stage) were subjected to two different analyses: one for a qPCR Ct threshold of ≤36 and one for a Ct threshold of ≤40. Very little is known about the pathology of Las infections, and there are some data that suggest that low titers (typically with Ct values greater than 36) detected in the psyllid may not always result in transmission of sufficient numbers of Las cells to initiate disease in citrus following ACP feeding [26] (Dr. Greg McCollum, ARS-USDA. personal communication, and personal observations in our laboratory). Therefore, we compared the data on the proportion of infected psyllids in two ways: PCR-positive psyllids with Ct values of ≤36, and those with Ct value of ≤40 which include those psyllids with low infection that may not be able to transmit Las in a manner that causes disease to citrus plants. However, means and SEMs of Ct values of all infected (qPCR-positive) psyllids are presented in S1 Table.

All proportions data were arcsine-transformed for the statistical analyses [31]. All statistical tests were conducted at the 0.05 or higher levels of significance. Additionally, for χ^2^ analysis on these proportions, results from the two replicated experiments for each life stage (nymphs or adults) were pooled for the sake of analyzing larger samples of psyllids in each sampling date. χ^2^ analysis was also used to analyze the proportion of Las-infected (PCR-positive) citrus seedlings or excised leaves that had been inoculated by feeding Las-exposed *D*. *citri* on them for 7 days at the consecutive Las-psyllid inoculativity tests described above.

#### Changes in Las titer with time in *D*. *citri*

Two-way ANOVA was performed using Graphpad version 6 (http://www.graphpad.com/guides/prism/6/statistics/index.htm?stat_holms_multiple_comparison_test.htm) to test the effects of life stage (nymphs or adults), acquisition time (1, 7 or 14 day AAP) and time post-acquisition (days padp) and their interactions on Las titer in *D*. *citri* (relative to that of the RPS20 psyllid gene). Additionally, Holm-Sidak multiple comparison test was used to determine the significance of variation in Las titer between 1, 7 or 14 day padp and all other padp measurements for nymphs and adults respectively, using α = 0.05. This same analysis was used to determine significance of variation between consecutive padp measurements (1 vs. 7 days, 7 vs. 14 days, etc.) for each AAP treatment and life stage. In our calculations of the increase in relative Las titer in *D*. *citri* over time, all the psyllids tested in each treatment/date (life stage, AAP, padp) were taken into consideration (including Las-negative psyllids with zero values).

## Results

### Las Acquisition by Nymphs and Adults of *D*. *citri*

Following acquisition access periods (AAPs) of 1 or 7 days for nymphs and 1, 7 or 14 days for adults on Las-infected citrus, the proportions of Las-infected (PCR-positive) psyllids were determined by qPCR on psyllids collected 4h to 1 day after the AAP and continued weekly for 5–6 weeks post-first access to diseased plants (padp) (Table 1). As mentioned earlier, all tested nymphs became adults at 7–14 days papd. ANOVA on the results of four experiments (2/life stage) indicated that the effect of life stage during Las-acquisition was highly significant whether Ct threshold values considered were ≤ 36 or ≤40 (Table 2). Additionally, with the adults only, AAP and the interaction of AAP with time post-acquisition (padp) were significant only when Ct threshold value was ≤40. No other effects or interactions were detected with nymphs or adults with either Ct value threshold used (Table 2).

**Table 1:** Las acquisition by *D*. *citri* nymphs and adults: Proportion of Las-infected (qPCR-positive) *D*. *citri* that had fed as nymphs or adults on Las-infected citrus plants for an acquisition access period (AAP) of 1, 7 or 14 days then transferred weekly to healthy citrus or orange jasmine seedlings^1^.

Stage at start of AAP	Days padp^2^	1-day AAP				7-day AAP				14-day AAP			
		Ct≤36		Ct≤40		Ct≤36		Ct≤40		Ct≤36		Ct≤40	
		No.	%	No.	%	No.	%	No.	%	No.	%	No.	%
**Nymphs**^**3**^	1–2	11/29	37.9	14/29	48.3	- ^4^	-	-	-	-	-	-	-
	7–8	13/36	36.1	18/36	50.0	16/34	47.1	19/34	55.9	-	-	-	-
	14	21/31	67.7	21/31	67.7	25/32	78.1	27/32	84.4	-	-	-	-
	21	9/32	28.1	11/32	34.4	12/33	36.4	13/33	39.4	-	-	-	-
	28	13/24	54.2	15/24	62.5	13/34	38.2	16/34	47.1	-	-	-	-
	35	26/38	68.4	28/38	73.7	24/43	55.8	29/43	67.4	-	-	-	-
	**Total**	**93/190**	**48.9**	**107/190**	**56.3**	**90/176**	**51.1**	**104/176**	**59.1**	-	-	-	-
**Adults**	1–2	5/64	7.8	10/64	15.6	-	-	-	-	-	-	-	-
	7–8	2/32	6.3	4/32	12.5	9/65	13.8	15/65	23.1	-	-	-	-
	14–15	0/29	0	0/29	0	4/32	12.5	8/32	25.0	18/61	29.5	21/61	34.4
	21	6/32	18.8	6/32	18.8	8/32	25.0	8/32	25.0	5/25	20.0	5/25	20.0
	28	1/12	8.3	2/12	16.7	4/17	23.5	7/17	41.2	3/32	9.4	4/32	12.5
	35	-	-	-	-	3/13	23.1	3/13	23.1	15/38	39.5	19/38	50.0
	42	-	-	-	-	-	-	-	-	1/14	7.1	1/14	7.1
	**Total**	**14/169**	**8.3**	**22/169**	**13.0**	**28/159**	**17.6**	**41/159**	**25.7**	**42/170**	**24.7**	**50/170**	**29.4**

^1^ Pooled results from qPCR analysis of two experiments for each life stage, with Ct value thresholds of ≤36 or ≤40.

^2^ padp = post-first access to diseased plants.

^3^All tested nymphs became adults 7–14 days padp.

^4^- = not done.

**Table 2:** ANOVA on the proportion of *D*. *citri* testing Las-positive by qPCR (analyses done on arcsine-transformed percentages)^1^.

Stage during AAP	Source of Variation^2^	Ct threshold ≤36			Ct threshold ≤40		
		*F*	*P*	Source df, error df	*F*	*P*	Source df, error df
**Nymphs**	AAP	0.1	0.76	1, 10	0.2	0.66	1, 10
	Days padp	0.3	0.91	5, 10	0.3	0.93	5, 10
	AAP x Days padp	0.3	0.90	4, 10	0.3	0.88	4, 10
**Adults**	AAP	1.8	0.20	2, 13	4.7	0.03*	2, 13
	Days padp	0.6	0.74	6, 13	1.6	0.21	6, 13
	AAP x Days padp	1.2	0.37	7, 13	3.5	0.03*	7, 13
**Over all**	AAP	0.9	0.43	2, 23	2.8	0.08	2, 23
	Days padp	1.1	0.39	7, 23	1.6	0.19	7, 23
	Stage	7.0	0.01**	1, 23	14.2	0.001***	1, 23
	AAP x Days padp	0.3	0.98	8, 23	0.6	0.73	8, 23
	AAP x Stage	0.1	0.79	1, 23	0.5	0.50	1, 23
	Days padp x Stage	0.5	0.74	4, 23	1.1	0.36	4, 23
	AAP x stage x Days padp	0.4	0.75	3, 23	0.6	0.60	3, 23

^**1**^ Results of two experiments / life stage.

^**2**^ AAP = acquisition access period, padp = post-first access to diseased plants.

* Significant at <0.05 level.

** Significant at 0.01 level.

*** Significant at 0.001 level.

Combined results of all four experiments (Table 1) show that, over the entire period of time post-acquisition tested (1–35 or 42 days padp), much higher proportions of psyllids that had access to infected plants as nymphs tested Las-positive (48.9–59.1%) compared to those that had access to infected plants as adults (8.3–29.4%). The difference in this regard between acquisition as nymphs or adults was highly significant at 1 or 7 days of AAP regardless of the Ct value threshold used (Table 3). Between 1 and 35 days padp, following AAPs as nymphs, the proportion of Las-exposed psyllids that tested Las-positive fluctuated between 34.4 and 73.7% with 1-day AAP, and between 39.4–84.4% with 7-day AAP, with no significant difference between these two AAPs (Table 3). However, with AAPs as adults, significant differences were found between 1 and 7 day AAPs at both Ct threshold values (≤ 36 or 40), and between 7 and 14 days only at Ct threshold of ≤ 36 (Table 3). The highest percentages of Las-infected psyllids that acquired Las as adults occurred at 21, 28 and 35 days padp, following AAPs of 1, 7 and 14 days respectively (Table 1). A slight to sharp decline in the percentage of Las-infected adults occurred at 28–42 days padp, but no such decline was observed near the end of the test period (on days 28 or 35 padp) with psyllids that acquired Las as nymphs (Table 1). In the control treatment (non-Las exposed psyllids) only 1.13% (3/265) of the tested psyllids were Las positive in qPCR tests. This very low infection rate may have been due to contamination or mislabeled PCR wells.

**Table 3:** χ^2^ analysis of Las acquisition results (data in Table 1).

Comparison between^1^	Other parameters	Ct value	χ^2^	*P*
**Nymphs vs. Adults**	**1-day AAP**	Ct≤36	70.69	0.000***
		Ct≤40	72.84	0.000***
	**7-day AAP**	Ct≤36	46.47	0.000***
		Ct≤40	42.36	0.000***
**1-day vs. 7-day AAP**	**Nymphs**	Ct≤36	0.18	0.676
		Ct≤40	0.29	0.591
	**Adults**	Ct≤36	4.33	0.037*
		Ct≤40	6.49	0.011**
**7-day vs. 14-day AAP**	**Adults**	Ct≤36	4.9	0.043*
		Ct≤40	1.27	0.259

^1^ AAP = acquisition access period.

* Significant at <0.05 level.

** Significant at 0.01 level.

*** Significant at 0.001 level.

The means and SEMs of Ct values of infected (qPCR-positive) psyllids 1–35 days padp are shown in S1 Table. When exposed as nymphs, mean Ct values of infected psyllids ranged from 26.8 ± 0.8 to 34.1± 0.8, whereas in those exposed as adults Ct values ranged from 28.5 ± 3.3 to 36.5 ± 0.3 (S1 Table). The relationship between Ct values and time padp in infected psyllids was significantly linear with nymphs that had 1-day AAP (intercept = 32.8 ± 0.67, slope = -0.06 ±0.03, P <0.05), and with adults that had either 1-day AAP (intercept = 36.3 ± 0.96, slope = 0.19±0.07, P <0.01) or 7-day AAP (intercept = 38.5±1.03, slope = -0.27±0.06, P .0001) (S2 Table). Significant differences in the intercept (*t* = 3.7, *P*<0.0003, df = 133) and the slope (*t* = 3.98, *P*<0.0001, df = 133) were found between nymphs and adults with 1-day AAP.

### Changes in Las Titer within *D*. *citri* following Acquisition by Nymphs or Adults

**Fig 1:**
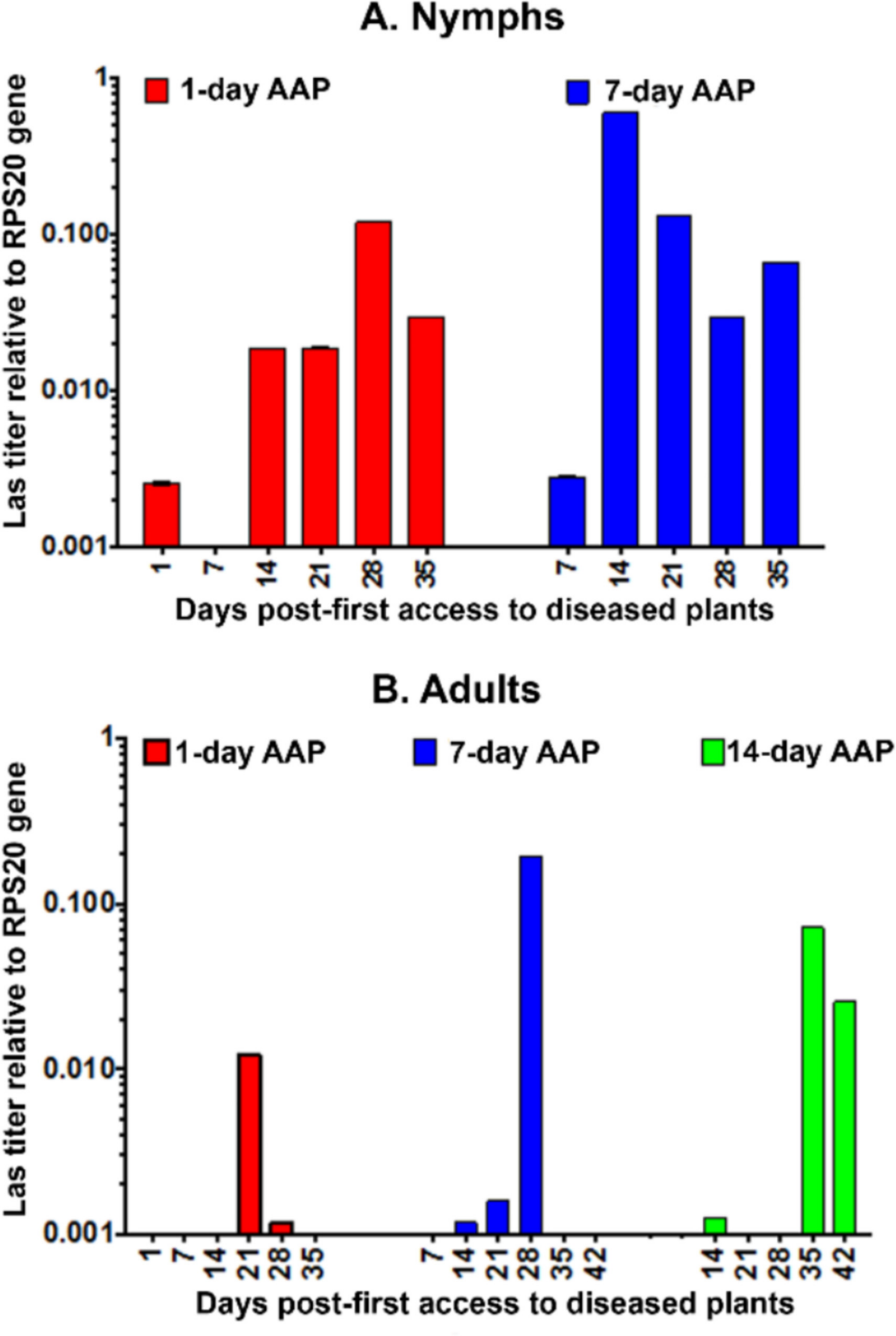
Las titer (Log scale) in *D*. *citri*, relative to that of the RPS20 psyllid gene, at various times following the start of 1, 7 or 14-day acquisition access periods (AAP) on Las-infected citrus plants as nymphs (A) or adults (B).

Las titer (Las genomes relative to those of the RPS20 psyllid gene) in *D*. *citri* individuals was monitored using qPCR analysis up to 35–42 days padp following the start of 1, 7 or 14 days of AAP as nymphs or as adults (Fig 1, S3 and S4 Tables). However, as mentioned earlier, all tested nymphs became adults at 7–14 days padp. ANOVA indicated that the effects of the length of acquisition period (AAP), time-post acquisition (padp) and their interaction on Las titer in *D*. *citri* were highly significant with both nymphs and adults (Table 4). Similarly, ANOVA indicated that the effects of life stage, time post-acquisition (padp) and their interaction on Las titer in *D*. *citri* were also highly significant (Table 5).

**Table 4:** ANOVA on the effects of acquisition access period (AAP), days post-first access to diseased plants (padp), and their interaction on Las titer (relative to RPS20 psyllid gene) following Las acquisition from infected citrus by *D*. *citri* nymphs or adults.

**Life stage**	**Source of variation**	**% of variation**	***F***	***P***
**Nymphs**	AAP	16.07	1.55E+07	0.0001
	Days padp	37.45	1.21E+07	0.0001
	AAP x Days padp	46.48	1.50E+07	0.0001
**Adults**	AAP	17.48	1.64E+07	0.0001
	Days padp	26.42	1.65E+07	0.0001
	AAP x Days padp	56.1	1.75E+07	0.0001

**Table 5:** ANOVA on the effects of life stage during the acquisition access period (nymphs vs. adults), days post-first access to diseased plants (padp) and their interaction on Las titer in *D*. *citri* (relative to RPS20 psyllid gene).

**Source of variation**	**% of variation**	***F***	***P***
**Life stage**	12.27	9.09E+06	0.0001
**Days padp**	35.18	5.21E+06	0.0001
**Stage x Days padp**	52.55	7.79E+06	0.0001

Following 1-day AAP by nymphs, Las titer increased gradually and significantly between 1 and 28 days padp. A much higher and faster increase in Las titer occurred between days 7 and 14 padp with nymphs that had a 7-day AAP (Fig 1A, S3 Table). In the 1-day and 7-day AAP treatments with nymphs, Las titer reached a peak at 28 and 14 days padp respectively, then it decreased slightly or fluctuated until 35 days padp. However, in psyllids that had access to infected plants as nymphs, Las titer was still significantly higher on day 35 than on days 1 or 7 padp whether AAP was 1 or 7 days (Fig 1A).

With *D*. *citri* that had access to infected plants as adults, Las titer increased at a slower rate and reached a much lower level compared to psyllids that had access to infected plants as nymphs (Fig 1A and 1B). However, this increase in Las titer was still significant between 1 and 21 days padp with 1-day AAP, between 7 and 14–28 days padp with 7-day AAP, and between 14 and 35–42 days padp with 14-day AAP (S4 Table). With psyllids that had an AAP as adults, the relative Las titer reached its peak at 21 days padp with 1-day AAP, 28 days padp with 7-day AAP, and 35 days padp with 14-day AAP. In the 1- and 7-dayAAP treatments by adults, Las titer declined significantly near the end of the test period between 28 and 42 days padp (Fig 1B). However, with 14-day AAP as adults Las titer was still significantly higher on days 35 and 42 padp than that on day14 padp.

### Las Inoculation by *D*. *citri* following Acquisition by Nymphs or Adults

Following AAPs of 1 or 7 days as nymphs, and 1, 7 or 14 days as adults, these psyllids were tested weekly for Las inoculation/transmission using sweet orange seedlings or excised leaves by caging 5–6 psyllids per seedling or excised leaf for 7 days, then testing the inoculated excised leaves one week later by qPCR, or sampling the inoculated seedlings 3, 6 and 9–12 months later for qPCR (3 leaves/plant/sampling date). As indicated earlier, all the nymphs used for Las acquisition became adults 7–14 days padp. The results using both methods (seedlings or excised leaves) indicated the ability of *D*. *citri* psyllids to inoculate/transmit Las when the AAP occurred during the nymphal stage but not when AAP occurred during the adult stage (Tables 6 and 7). A total of 119 sweet orange seedlings and 90 excised leaves inoculated by *D*. *citri* that fed on Las-infected plants as adults proved Las-negative up to 9–12 months later, despite the fact that 8.3–29.4% of these adults proved Las-positive by qPCR (Tables 1 and 6). However, with *D*. *citri* that had access to Las-infected plants as nymphs, 5.2% of the inoculated excised leaves (N = 116) and 20.8% of the inoculated seedlings (N = 53) proved Las-positive (Table 6) over the test period (7–35 days padp). The difference in Las-inoculativity between psyllids that acquired Las as nymphs or adults is significant whether seedlings or excised leaves were used for inoculation (Table 7). Also, psyllids that acquired this bacterium as nymphs for 7 days infected significantly more plants than did those that acquired for only 1 day (Table 7). The average Ct values for Las positive inoculated seedlings and excised leaves, respectively, were 29.4 and 36.7. In the negative control treatment, 17 leaves and 12 seedlings inoculated by non Las-exposed psyllids (5–6 psyllids/leaf or seedling) were similarly processed for qPCR and all proved Las-negative.

**Table 6:** Las inoculation/transmission by psyllids: Results of inoculativity tests on *D*. *citri* that fed as nymphs or adults on Las-infected citrus plants for an acquisition access period (AAP) of 1, 7 or 14 days, then tested weekly for Las inoculation into healthy sweet orange seedlings or excised leaves (5–6 insects/ seedling or excised leaf) at weeks 2–5 post-acqusition^1^.

**Stage at start of AAP**	Test type (leaves/ seedlings)^2^	Days padp^3^	1-day AAP^4^	**7-day AAP**	**14-day AAP**	**Total**	
						**No.**	**%**
**Nymphs**	**Leaves**	**7–14**	1/20	2/20	- ^5^	3/40	7.50
		**14–21**	0/17	0/20	-	0/37	0.00
		**21–28**	3/17	0/16	-	3/33	9.09
		**28–35**	0/2	0/4	-	0/6	0.00
		**Overall**	**4/56**	**2/60**	**-**	**6/116**	**5.17**
	**Seedlings**	**7–14**	0/7	1/10	-	1/17	5.88
		**14–21**	1/4	5/6	-	6/10	60.00
		**21–28**	0/6	4/12	-	4/18	22.22
		**28–35**	0/2	0/6	-	0/8	0.00
		**Overall**	**1/19**	**10/34**	**-**	**11/53**	**20.75**
**Adults**	**Leaves**	**7–14**	-	-	-	-	-
		**14–21**	0/10	0/10	0/10	0/30	0.00
		**21–28**	0/10	0/10	0/10	0/30	0.00
		**28–35**	0/10	0/10	0/10	0/30	0.00
		**Overall**	**0/30**	**0/30**	**0/30**	**0/90**	**0.00**
	**Seedlings**	**7–14**	0/17	0/14	0/5	0/31	0.00
		**14–21**	0/12	0/11	0/4	0/28	0.00
		**21–28**	0/6	0/9	0/11	0/19	0.00
		**28–35**	0/14	0/16	-	0/41	0.00
		**Overall**	**0/49**	**0/50**	**0/20**	**0/119**	**0.00**

^1^Pooled results from two experiments for each life stage.

^2^Healthy young excised leaves or seedlings of sweet orange were inoculated using 5–6 Las-exposed psyllids/leaf or seedling for 7 days. Leaves were then incubated at 25°C for one week before being processed for qPCR, whereas inoculated seedlings were placed in the greenhouse and sampled for qPCR (3 leaves/plant) every three months until 9–12 months post inoculation. For controls, 17 leaves and 12 seedlings inoculated by non-infected psyllids were similarly processed for qPCR and all proved Las-negative.

^3^Padp = post first-access to diseased plants.

^4^Plants or leaves tested at 1–7 days padp from the 1-day AAP treatment (all proved Las-negative) were not included in this table because this period may correspond with the latent period for Las in *D*. *citri* (as explained in the discussion).

^5^- = test not done.

**Table 7:** χ^2^ analysis of inoculativity test results (data in Table 6).

Comparison between^1^	**Other parameters**	χ^2^	*P*
Nymphs vs. adults	Leaves	4.795	0.029*
	Seedlings	26.386	0.000***
1-day vs. 7-day AAP	Nymphs on leaves	0.857	0.355
	Nymphs on seedlings	4.322	0.038*
Leaves vs. seedlings	Nymphs	9.763	0.002**

^**1**^AAP = acquisition access period.

* Significant at <0.05 level.

** Significant at 0.01 level.

*** Significant at 0.001 level.

With *D*. *citri* that were given a 1-day AAP as nymphs, no Las-inoculation/ transmission was obtained before 7 days padp; all 12 leaves and 10 seedlings inoculated during 1–7 days padp (5–6 insects/leaf or seedling) proved Las-negative later by qPCR. This early test period (1–7 days padp) may represent a latent period for the bacteria in the vector (as suggested later in the discussion). However, *D*. *citri* that were given either 1- or 7-day AAP as nymphs were able to inoculate Las into sweet orange seedlings or excised leaves starting with 7–14 days padp and continued transmitting this bacterium up to 28 days padp (Table 6). The highest inoculation rates obtained by these psyllids occurred at 14–28 days for inoculated seedlings (22.2–60.0%) and at 21–28 days padp for excised leaves (9.09%) (Table 6). The highest inoculation/transmission success rate was observed (using the seedling assay) at 14–21 days padp with psyllids that had a 7-day AAP as nymphs. These psyllids were also shown to have the highest Las titer as indicated earlier (Fig 1A).

## Discussion

Previous investigations on the transmission mechanism of Las by its psyllid vector, *D*. *citri*, indicated that Las is a circulative pathogen, i.e. passes through the molting process from one nymphal instar to the next and from nymphs to adults, and persistent, i.e. once acquired by nymphs or adults it remains in the vector for a long time, sometimes for life [3,10,11,15,20]. However, previous investigations on Las-vector relations gave somewhat conflicting evidence on whether Las multiplies in *D*. *citri*, i.e. whether Las can be considered propagative or non-propagative in its vector [3, 20]. Indirect evidence used to suggest that Las does not multiply in the vector includes reports that the percentage of Las-infected (PCR-positive) psyllids decreased with time post-acquisition by nymphs [16], and that the efficiency of Las transmission or inoculation into healthy plants by Las-exposed psyllids is very low [3,16,20,26]. On the other hand, indirect evidence suggesting that Las multiplies in *D*. *citri* included the following: a. adult *D*. *citri* can remain inoculative with Las through most of their life span after acquiring this bacterium as nymphs [15], b. almost systemic infection of Las in the vector following Las acquisition [13,14,17], and c. qPCR results showing higher Las titer in *D*. *citri*’s alimentary canal and salivary glands compared to other tissues [14]. The only direct evidence of Las replication in *D*. *citri*, so far, was reported by Inoue et al. [18], who found that Las titer (as estimated by qPCR) significantly increased on days 10–20 post-acquisition by nymphs, but not following acquisition by adults. Our results confirm and extend those by Inoue et al. [18] who used only 1-day AAP and tested Las titer until 20 days post-acquisition, whereas we tested AAPs of 1–14 days and continued our qPCR testing of the psyllids up to 35–42 days padp.

In our present work, linear regression analysis indicated that Ct values decreased significantly (suggesting higher Las titers) with time between 1–35 days padp in nymphs and adults that had 1-day AAP on diseased plants. Furthermore, Las titer (relative to that of the RPS20 psyllid gene) also increased significantly in both nymphs and adults of *D*. *citri* post-acquisition, but it increased much faster and attained a much higher level when acquired by nymphs than when acquired by adults (Fig 1). In the adults, it took at least 21–35 days padp to reach a significant increase in Las titer following 1–14 days AAP, whereas in nymphs a significant increase in Las titer occurred 14 days padp with either 1- or 7 day AAP. Our results strongly suggest that Las multiplies in *D*. *citri* whether acquired by nymphs or adults but its titer increases much faster when acquired by nymphs than by adults, and that adults may require longer AAP on infected plants compared to nymphs for Las to reach appreciably higher levels in the vector. Additionally, regardless of the AAP tested, the rate of Las acquisition (proportion of Las-exposed psyllids that proved Las-positive by qPCR) was significantly higher when Las was acquired by nymphs than by adults, which is consistent with previous investigations [16,18]. The reasons why Las appears to multiply faster or more efficiently in nymphs than in adults, as shown by us and by Inoue et al. [18], may be due to one or several factors that should be investigated further. These factors may include: a. different feeding behaviors in psyllid nymphs than in adults, e.g. it is known that nymphs of *D*. *citri* feed on younger citrus tissues than do the adults [3,12,20]; b. Las transmission barriers or binding sites in the gut or salivary glands [13,14,21] may be different in psyllid nymphs than in adults; c. the symbiotic organisms found in the nymphs may be different than those found in the adults or may affect Las replication differently in both life stages.

Under the conditions of our experiments, only psyllids that had access to infected plants during the nymphal stage were able to inoculate Las into healthy citrus seedlings or excised leaves whereas those that acquired Las as adults did not, regardless of the length of the AAP used. These results suggest that multiplication of Las bacterium to a certain threshold level within the psyllids is essential for efficient transmission, and that such threshold can only be reached if Las was acquired by *D*. *citri* during the nymphal rather than the adult stage, which is consistent with previous results from Japan. [18]. It is also possible that multiplication of Las acquired by the adults is too slow to reach the salivary glands in a high enough titer in time to be transmitted by these adults which could be near the end of their life span. In Brazil, however, Colletta-Filho et al. [32] reported a very low inoculation rate (0.7%) for *D*. *citri* that acquired Las during the adult stage (acquisition and transmission rates by nymphs were not tested). Thus, the main conclusion from the three investigations studying Las transmission/inoculation efficiency by *D*. *citri* (Inoue et. al. [18], Colletta-Filho et al. [32] and our current work) is that a significantly higher proportion of psyllids can inoculate Las into citrus when these psyllids have acquired Las from infected plants during the nymphal rather than the adult stage. This conclusion has significant implications in the epidemiology and control of this economically important citrus disease. For example, chemical control measures against *D*. *citri* should be targeted to young citrus flush, which is usually preferred by psyllid nymphs compared to older tissue [2,3].

It is not known whether the results obtained here with *D*. *citri* populations from Florida can be generalized to other Las/HLB vectors (e.g. *Trioza erytreae* vector of HLB pathogen in Africa) or to other *D*. *citri* and Las populations in other parts of the world, since acquisition and transmission characteristics of various pathogens may be different in different pathogen and/or vector populations probably due to genetic/environmental variations [3,21,23]. Interestingly, higher transmission/inoculation rates of Las by *D*. *citri* were reported in China [15] and Japan [18] than in Florida [16,26 and this work]. Although Xu et al [15] and Inoue et al. [18] used much smaller numbers of test plants for Las inoculation than we did, it is possible that Chinese and/or Japanese populations of the vector or the pathogen may be different than the Florida populations in vector competence/ transmission efficiency. Additionally, differences in the experimental procedure or conditions may affect the results of inoculation tests in different labs or in different parts of the world.

In our work, *D*. *citri* nymphs exposed to Las infected plants for 1 day, were able to inoculate this bacterium into citrus leaves or seedlings at 7–14 days padp, but not before that. A latent period of 1–25 days for Las in *D*. *citri* has been reported [15]. For circulative and propagative pathogens, the latent period is usually necessary for the pathogen to multiply and/or to cross the vector’s midgut and reach the salivary glands before transmission/inoculation can take place [21,22,23]. Our results suggest that when *D*. *citri* nymphs have an AAP of 1 day the latent period can be a minimum of 7 days. However, a shorter latent period of Las in psyllid nymphs may be possible with longer AAPs, but it would be difficult to demonstrate since it might overlap with the longer AAP. Based on the slower multiplication of Las in *D*. *citri* adults, it is also possible that much longer latent periods in the vector may be required when Las is acquired by adults, which may explain why we did not get any transmission/inoculation up to 35 days padp by psyllids that acquired Las as adults even with a 14-day AAP. The highest rates of Las inoculation (into citrus seedlings) were obtained at 14–28 days padp by psyllids that acquired Las as nymphs, which corresponds with the period of highest Las titers in these psyllids (Table 6, Fig 1A). This also means that by this time Las must have entered and probably multiplied in the salivary glands as we suggested earlier [13,14]. With another phloem-limited psyllid-borne bacterium, *Candidatus* Liberibacter solanacearum (Lso), the putative causal agent of zebra chip disease in potatoes and tomatoes transmitted by *Bactericera cockerelli*, a minimum of 2 week latent period in the vector has been reported [33,34]. Lso titer in *B*. *cockerelli* peaked two weeks post-acquisition and psyllid infectivity (inoculativity) was associated with Lso colonization of insect salivary glands and with Lso copy numbers of 10,000 per psyllid [33]. Because Lso is much more efficiently transmitted by *B*. *cockerelli* than Las by *D*. *citri*, Sengoda et al. [34] tested Lso-exposed psyllids singly for inoculation into host plants, whereas we used groups of 5–6 psyllids/plant or leaf for inoculation. We also used both excised leaves and citrus seedlings for Las inoculation by *D*. *citri*, and we obtained higher percentages of inoculation with seedlings than with excised leaves, but the latter can still provide a much faster and more convenient way of testing Las inoculativity by psyllids compared to whole plants [26]. In a recent study on Las and *D*. *citri* from Japan, 8–9% of the psyllids collected from field-infected trees were infective to citrus seedlings [17]. In that study, it was estimated that the Las titer threshold for *D*. *citri* inoculativity was around 10^6^ Las copies per psyllid, which is much higher than the threshold estimated for Lso in *B*. *cockerelli* [33]. This difference in titer threshold for inoculativity may partly explain why *B*. *cockerelli* is a much more efficient vector of Lso than *D*. *citri* is for Las. It is also possible that this difference in psyllid inoculativity titer between the two pathogens reflects differences in feeding behavior between the two vectors, and/or that citrus plants require larger doses of Las during its inoculation by psyllids to get infected compared to the doses required for potato or tomato plants in relation to Lso infection. The fact that citrus is a woody (perennial) tree whereas tomatoes and potatoes are herbaceous (annual) plants may explain this difference in the required pathogen dose. That a very high percentage of citrus trees still get infected in the field by Las, in spite of the relatively low vector efficiency of *D*. *citri*, is probably due to the huge numbers of Las-infected *D*. *citri* that are usually found on these trees in Florida and other places recently invaded by Las and its vector, to the persistence of Las in the vector, and to the mobility of infected psyllid adults [2,35].

In both Las and Lso pathosystems, the increase in exposure time of psyllids to infected plants generally increases the bacterial titer in the vector [33, and present work]. This is possibly due to an increase in the bacterial inoculum/dose ingested by the psyllids with longer AAP’s, which may explain why a much higher increase in Las titer within *D*. *citri* adults occurred with a 7 or 14-day AAP, compared to 1-day AAP. However, the effect of the length of AAP seems much less important with *D*. *citri* that acquired Las as nymphs, where both 1 and 7 day AAP resulted in significant increase in Las titer at 14 days padp, although Las titer attained at 7 days padp was not significantly different between 1- and 7- day AAP. The apparent decline and/or fluctuation in Las titer in *D*. *citri* after 14–35 days post acquisition, also reported by Inoue et al. [18] and with several other vector-born plant and animal viruses [21,22,23], may be interpreted in two, nonexclusive, ways: 1, that Las replication is slower or less efficient in older psyllids than in younger ones, which is supported by the findings that Las multiplies faster and to higher levels when acquired by nymphs than when acquired by adults [18, and present work], and/or 2, that the rate of Las discharge from the vector’s salivary glands during feeding/inoculation may be faster than can be replenished by Las replication especially in older adults. These two possibilities may also explain the intermittent inoculation or transmission of Las by single or small groups of *D*. *citri* in serial inoculation tests on host plants [15,17,26], and the decrease in the percentage of infected psyllids with time post-acquisition [16]. However, the latter result can also be explained if the bacterium reduces the longevity of the psyllid vector as recently reported [36], which may lead to lower rates of infected psyllids as they grow older.

Additionally, the role of the insect vector’s immune system in suppressing the multiplication of plant pathogens in their vectors has been demonstrated in some cases, e.g. with tomato yellow leaf curl virus in its whitefly vector [37,38], and this factor should be investigated in future studies on other pathogen-vector systems including Las, Lso and their psyllid vectors. Another related point that needs further investigation is the possibility that the much higher proportion of seemingly Las-infected (PCR-positive) *D*. *citri*, compared to those that can inoculate the pathogen into host plants, could be partly the result of PCR detecting DNA from both live and dead Las cells in the infected psyllids. However, for the purpose of our study, the fact that we detected a significant increase in Las DNA over time post-acquisition in psyllids means that the bacterium must have replicated to produce that DNA, regardless of whether the bacterial cells subsequently died or not. Despite this distinction, understanding Las replication and longevity in the psyllids, as related to psyllid age, innate immunity and/or to other bacterial inhabitants of the psyllid (endosymbiotes, etc.), is of great interest and deserves further investigation.

In the Lso-*B*. *cockerelli* system, psyllid adults are reported to be more efficient than nymphs in acquiring and transmitting Lso from/into host plants [39]. Furthermore, Lso multiplies efficiently in *B*. *cockerelli* adults, but it reaches a plateau around 15 days post acquisition up to 20 days [33]. In the present work, we found that Las titer increased in *D*. *citri* nymphs much faster than in adults, but it reached a peak around 14–28 days following acquisition by nymphs and 21–35 days following acquisition by adults, after which it declined or fluctuated. This is much longer than Lso was tested in *B*. *cockerelli* [33]. The two systems (Las and Lso) are similar in being circulative and propagative in their psyllid vectors, but apparently have a fundamental difference in that nymphs are much more efficient than adults in acquisition and inoculation of Las by *D*. *citri*, whereas the opposite was reported to be the case with Lso and *B*. *cockerelli* [39,40]. In most other pathogen-vector systems investigated so far (in the circulative/propagative associations) nymphs are usually more efficient in acquisition and/or transmission than adults [21,23]. Additionally, transmission/inoculation efficiency by single psyllids, is generally much higher in the Lso- *B*. *cockerelli* system using nymphs or adults for acquisition (21–79%) compared to that of Las-*D*. *citri* system, mainly using nymphs for acquisition (mostly 1–12%) [16,17,26,39]. Interestingly, using FISH, Lso was found in 66% of the alimentary canals and 39% of the salivary glands of *B*. *cockerelli*, whereas Las was found only in 44% of the alimentary canals and 21% of the salivary glands of *D*. *citri* [13,40]. The above results may indicate that entry or exit transmission barriers found in the midgut and/or salivary glands are more restrictive in *D*. *citri* adults than in nymphs, and also more restrictive in the Las-*D*. *citri* system than in the Lso- *B*. *cockerelli* one.

Transmission barriers, especially related to the midgut and salivary glands have been reported or suggested earlier as major determinants of vector specificity and vector competence in many vector-borne plant and animal bacterial, viral and other pathogens [14,21,22,23,41,42]. Novel control strategies using vector transgenesis that aim at reducing insect vectorial capacity, or seek to eliminate transmission of pathogens such as pea enation mosaic virus, *Plasmodium* sp., *Trypanosoma* sp., and Dengue virus are currently being developed [43,44]. Further elucidation of barriers to acquisition, replication and inoculation of Las and Lso in their respective psyllid vectors could provide information necessary to devise new strategies to protect citrus, potatoes, tomatoes and other important crops from these fairly recent and economically serious pathogens.

## Supporting Information

S1 Table Means ± SEMs of Ct values in weekly qPCR tests of *D*. *citri* that fed on Las-infected plants as nymphs or adults for an acquisition access period (AAP) of 1, 7 or 14 days. (DOCX)

S2 Table Results of regression analyses on Ct values in qPCR tests of *D*. *citri* that fed on Las-infected plants as nymphs or adults for an acquisition access period (AAP) of 1, 7 or 14 days. (DOCX)

S3 Table Las replication in *D*. *citri* following acquisition as nymphs: Multiple comparisons of post-acquisition differences in Las titer (relative to RPS20 psyllid gene) between 1 and 35 days post-first access to diseased plants (padp) in *D*. *citri* that were exposed as nymphs to Las-infected plants for 1- or 7-day acquisition access period (AAP). (DOCX)

S4 Table Las replication in *D*. *citri* following acquisition as adults: Multiple comparisons of post-acquisition differences in Las titer (relative to RPS20 psyllid gene) between 1 and 42 days post-first access to diseased plants (padp) in *D*. *citri* that were exposed as adults to Las-infected plants for 1-, 7- or 14-day acquisition access period (AAP). (DOCX)
