# Supplementary material for: Acquisition, Replication and Inoculation of Candidatus Liberibacter asiaticus following Various Acquisition Periods on Huanglongbing-Infected Citrus by Nymphs and Adults of the Asian Citrus Psyllid
Source: PLoS One. 2016 Jul 21;11(7):e0159594. doi: 10.1371/journal.pone.0159594 (PMC4956146; doi:10.1371/journal.pone.0159594)
Supplement: S1 Table — (DOCX) [file pone.0159594.s001.docx]

**Supporting Information**

| **S1 Table. Means ± SEMs of Ct values in weekly qPCR tests of *D. citri* that fed on Las-infected plants as nymphs or adults for an acquisition access period (AAP) of 1, 7 or 14 days** | | | | | | | |  |
| --- | --- | --- | --- | --- | --- | --- | --- | --- |
|  | | Days post-first access to diseased plants (padp) | | | | | | |
| Stage | AAP duration (days) | 1-2 | 7 | 14-15 | 21 | 28 | 35 | |
| Nymphs | 1 | 33.0±0.7 | 34.1±0.8 | 30.0±0.9 | 29.7±1.3 | 32.0±1.1 | 31.2±0.8 | |
|  | 7 | - | 32.9±0.6 | 26.8±0.8 | 31.3±0.9 | 32.9±0.9 | 30.8±0.9 | |
|  |  |  |  |  |  |  |  | |
| Adults | 1 | 36.2±0.8 | 35.2±1.4 | - | 30.3±1.0 | 34.9±3.8 | - | |
|  | 7 | - | 36.5±0.3 | 35.1±1.1 | 32.0±1.3 | 31.9±2.5 | 28.5±3.3 | |
|  | 14 | - | - | 33.9±0.8 | 30.7±1.1 | 35.4±1.2 | 32.6±2.3 | |
