# Supplementary material for: Acquisition, Replication and Inoculation of Candidatus Liberibacter asiaticus following Various Acquisition Periods on Huanglongbing-Infected Citrus by Nymphs and Adults of the Asian Citrus Psyllid
Source: PLoS One. 2016 Jul 21;11(7):e0159594. doi: 10.1371/journal.pone.0159594 (PMC4956146; doi:10.1371/journal.pone.0159594)
Supplement: S2 Table — (DOCX) [file pone.0159594.s002.docx]

**Supporting Information**

| **S2 Table. Results of regression analyses on Ct values in qPCR tests of *D. citri* that fed on Las-infected plants as nymphs or adults for an acquisition access period (AAP) of 1, 7 or 14 days** | | | | | | | |
| --- | --- | --- | --- | --- | --- | --- | --- |
| Stage | AAP (days) | *F*-value | *P* | Source df, error df | Intercept (SEM) | Slope (SEM) | r^2^ |
| Nymphs | 1 | 3.9 | 0.05 | 1, 113 | 32.8 (0.67) | -0.06 (0.03) | 0.03 |
|  | 7 | 0.7 | 0.40 | 1, 101 | - | - | 0.01 |
|  |  |  |  |  |  |  |  |
| Adults | 1 | 8.2 | 0.01 | 1, 20 | 36.3 (0.96) | -0.19 (0.07) | 0.29 |
|  | 7 | 21.8 | <0.0001 | 1, 45 | 38.5 (1.03) | -0.27 (0.06) | 0.33 |
|  | 14 | 0.2 | 0.64 | 1, 39 | - | - | 0.01 |
