# Supplementary material for: Acquisition, Replication and Inoculation of Candidatus Liberibacter asiaticus following Various Acquisition Periods on Huanglongbing-Infected Citrus by Nymphs and Adults of the Asian Citrus Psyllid
Source: PLoS One. 2016 Jul 21;11(7):e0159594. doi: 10.1371/journal.pone.0159594 (PMC4956146; doi:10.1371/journal.pone.0159594)
Supplement: S3 Table — (DOCX) [file pone.0159594.s003.docx]

**Supporting Information**

**S3 Table. Las replication in *D. citri* following acquisition as nymphs: Multiple comparisons of post-acquisition differences in Las titer (relative to RPS20 psyllid gene) between 1 and 35 days post-first access to diseased plants (padp) in *D. citri* that were exposed as nymphs to Las-infected plants for 1- or 7-day acquisition access period (AAP)^1^**

| **Days padp** |  | **1-day AAP** |  |  |  | **7-day AAP** | |  |
| --- | --- | --- | --- | --- | --- | --- | --- | --- |
|  | **Mean Las**  **Titer^2^** | **Mean**  **difference**  **with day 1** | **SE of**  **difference** | ***P*** | **Mean Las**  **Titer^2^** | **Mean difference with day 7** | **SE of difference** | ***P*** |
| **1** | 0.00256d | - | - | - | - | - | - | - |
| **7** | 0.00059e | -0.00198 | 0.000084 | 0.0001 | 0.00281e | - | - | - |
| **14** | 0.01890c | 0.01634 | 0.000082 | 0.0001 | **0.60230a** | 0.5994 | 0.000067 | 0.0001 |
| **21** | 0.01901c | 0.01644 | 0.000083 | 0.0001 | 0.13430b | 0.1315 | 0.000068 | 0.0001 |
| **28** | **0.12050a** | 0.11790 | 0.000085 | 0.0001 | 0.02941d | 0.0266 | 0.000066 | 0.0001 |
| **35** | 0.03003b | 0.02746 | 0.000081 | 0.0001 | 0.06694c | 0.0641 | 0.000065 | 0.0001 |

^1^ Holm-Sidak multiple comparisons test (α=0.05)

^2^Means followed by different letters within each column are significantly different; letter ‘a’ marks highest value in each AAP treatment (bold) followed by b, c, etc.
