# Supplementary material for: Acquisition, Replication and Inoculation of Candidatus Liberibacter asiaticus following Various Acquisition Periods on Huanglongbing-Infected Citrus by Nymphs and Adults of the Asian Citrus Psyllid
Source: PLoS One. 2016 Jul 21;11(7):e0159594. doi: 10.1371/journal.pone.0159594 (PMC4956146; doi:10.1371/journal.pone.0159594)
Supplement: S4 Table — (DOCX) [file pone.0159594.s004.docx]

**Supporting Information**

**S4 Table. Las replication in *D. citri* following acquisition as adults: Multiple comparisons of post-acquisition differences in Las titer (relative to RPS20 psyllid gene) between 1 and 42 days post-first access to diseased plants (padp) in *D. citri* that were exposed as adults to Las-infected plants for 1-, 7- or 14-day acquisition access period (AAP) ^1^**

| **Days padp** |  | **1-day AAP** | |  |  | **7-day AAP** | |  |  | **14-day AAP** | | |
| --- | --- | --- | --- | --- | --- | --- | --- | --- | --- | --- | --- | --- |
|  | **Mean Las**  **Titer^2^** | **Mean**  **differ.**  **with**  **day 1** | **SE of difference** | ***P*** | **Mean Las**  **Titer^2^** | **Mean**  **differ.**  **with**  **day 7** | **SE of difference** | ***P*** | **Mean Las**  **Titer^2^** | **Mean**  **differ.**  **with**  **day 14** | **SE of differ.** | ***P*** |
| **1** | 0.000006c | - | - | - | - | - | - | - | - | - | - | - |
| **7** | 0.000023c | 0.000017 | 0.000015 | 0.7309 | 0.000074d | - | - | - | - | - | - | - |
| **14** | 0.000000c | -0.000006 | 0.000016 | 0.9700 | 0.001174c | 0.001099 | 0.000016 | 0.0001 | 0.001242c | - | - | - |
| **21** | **0.012060a** | 0.012050 | 0.000016 | 0.0001 | 0.001588b | 0.001513 | 0.000015 | 0.0001 | 0.000638d | -0.000017 | 0.0021 | 0.0001 |
| **28** | 0.001185b | 0.001179 | 0.000022 | 0.0001 | **0.194400a** | 0.194400 | 0.000019 | 0.0001 | 5.106E-07e | -0.000018 | 0.0020 | 0.0001 |
| **35** | 0.000000c | -0.000007 | 0.000050 | 0.9895 | 0.000020e | -0.000055 | 0.000013 | 0.0001 | **0.07156a** | 0.000015 | 0.0024 | 0.0001 |
| **42** | - | - | - | - | 0.000024e | -0.000050 | 0.000014 | 0.0004 | 0.02557b | 0.000020 | - 1. .0.0023 | 0.0001 |

^1^ Holm-Sidak multiple comparisons test (α=0.05).

^2^Means followed by different letters within each column are significantly different; letter ‘a’ marks highest value in each AAP treatment (bold) followed by b, c, etc.
